# Data driven of underground water level using artificial intelligence hybrid algorithms

**DOI:** 10.1038/s41598-023-35255-9

**Published:** 2023-06-26

**Authors:** Mohammadtaghi Rahimi, Hossein Ebrahimi

**Affiliations:** 1 Department of Civil Engineering, Kish international Branch, Islamic Azad University, Kish Island, Iran; 2 Department of Water Science and Engineering, Shahr-e-Qods Branch, Islamic Azad University, Tehran, Iran

**Keywords:** Climate sciences, Environmental sciences

## Abstract

As the population grows, industry and agriculture have also developed and water resources require quantitative and qualitative management. Currently, the management of water resources is essential in the exploitation and development of these resources. For this reason, it is important to study water level fluctuations to check the amount of underground water storage. It is vital to study the level of underground water in Khuzestan province with a dry climate. The methods which exist for predicting and managing water resources are used in studies according to their strengths and weaknesses and according to the conditions. In recent years, artificial intelligence has been used extensively for groundwater resources worldwide. Since artificial intelligence models have provided good results in water resources up to now, in this study, the hybrid model of three new recombined methods including FF-KNN, ABC-KNN and DL-FF-KNN-ABC-MLP has been used to predict the underground water level in Khuzestan province (Qale-Tol area). The novelty of this technique is that it first does classification by presenting the first block (combination of FF-DWKNN algorithm) and predicts with the second block (combination of ABC-MLP algorithm). The algorithm’s ability to decrease data noise will be enabled by this feature. In order to predict this key and important parameter, a part of the data related to wells 1–5 has been used to build artificial intelligence hybrid models and also to test these models, and to check this model three wells 6–8 have been used for the development of these models. After checking the results, it is clear that the statistical RMSE values of this algorithm including test, train and total data are 0.0451, 0.0597 and 0.0701, respectively. According to the results presented in the table reports, the performance accuracy of DL-FF-KNN-ABC-MLP for predicting this key parameter is very high.

## Introduction

Underground water is one of the most important natural resources in desert and semi-desert countries. Underground water sources are used to provide water for industry, agriculture, and drinking water^1,2^. In fact, the primary source of water supply used in agriculture is underground water^3^. Consequently, excess extraction of these resources has become a major problem in recent years^4^. Underground water is dynamic and can adapt to short-term and long-term changes in weather conditions, groundwater extraction, and land use changes. Moreover, the balance between charging and discharging aquifers controls the level of underground water. Groundwater management is facing problems due to lack of water, irregular and indirect rainfall, as well as lack of surface water in desert and semi-desert regions^5^. Furthermore, the amount of groundwater is an integral part of groundwater management^6^. Reduction in underground water supply the overexploitation of these sources reduce the amount of groundwater over time, which causes problems. Problems include salinization of underground water^7^, integrating of salt water with fresh water, and the increased level of industrial pollutants^8^. Since it is difficult to check the level of underground water in large areas and also it is very difficult to use direct and field methods, the use of modern methods such as artificial intelligence algorithms are more efficient due to less cost and time^6,9,10^.

In 2020, Kumar et al. used ELM, GPR and DL to predict the underground water level in Japan. The data used in this study are precipitation, temperature, river flow, nutrition, and depth of underground water. The results obtained from this study showed that the model used in the study (RMSE = 0.04, R = 0.99, NSE = 0.98) is more accurate in predicting the level of underground water. Their report demonstrates that the DL model performs better on small datasets^11^. In the same year, Sahu et al. utilized various input variables such as river flow levels, temperature, groundwater, and precipitation to forecast the underground water level in California, USA. The study employed MLP and DL models to predict these algorithms. The findings revealed that DL models were effective in forecasting the underground water level^12^. Emamgholizadeh and Mohammadi presented a new hybrid method based on SVM, PSO, and IWO models with SVM-PSOIWO structure for estimating soil exchange capability (CEC). Based on the findings of this paper, it can be concluded that the novel combination algorithm, when applied to the prediction of a three-month period with RMSE (R2) of 0.229 Cmol + kg^−1^ (0.924), has a high degree of accuracy^13^. Vadiati et al., by FL, ANFIS and SVM predicted the underground water level in the Tehran Karaj plain. The data used in this study are: total rainfall, evaporation of groundwater, average temperature, and total transpiration and monthly average river flow. Their results have been shown ANFIS is highly accurate in prediction of underground water level, but all three methods used in this study have good performance. The models used in this study predict the underground water level for the next 1 and 2 months, and the prediction of these models for the next 3 months is also acceptable^14^. Mohammadi predicted Peru’s hydrological conditions over the course of 3, 6, and 24 months using the ANN-FA model. The standardized precipitation index (SPI) of the surrounding areas is used as input data in this study. The findings for this new approach, which have RMSE = 0.29 and R = 0.94, demonstrate the excellent level of performance accuracy of this algorithm. He noted that this model might also be useful in other areas^15^. In this study, 2112 data sets collected from 8 wells were used to predict the underground water level of Khuzestan region in Iran. To predict this important parameter, FF-KNN, ABC-KNN and DL-FF-KNN-ABC-MLP algorithms were used. A characteristic and abilities of this algorithm is high accuracy, high speed and good performance. The results show that the DL-FF-KNN-ABC-MLP algorithm has an accuracy of performance over the other algorithms introduced in this article.

## Materials and methods

### KNN algorithm

The KNN algorithm is one data mining algorithms that is primarily used in data classification. This algorithm finds k samples of the training data which are closer to the test sample than all the training data and calculates the average output of these k samples and considers them as the estimated final value for the test sample. The requirements of this algorithm include: First, we need to have a set of samples with output or labeled data, second, we need a similarity unit or distance to calculate the distance between two samples, and third, we need to specify a k value to determine the number of neighbors. KNN or WKNN algorithm is the same as KNN, with the difference that for each test sample, each sample from the k set that is obtained, according to how far it is from the test point, a coefficient is placed for that sample to Those that have a greater distance have less effect on the output and closer samples have a greater effect^16^. First, the distance from the test sample to all training samples is calculated using Eq. (1):

1 $${D}_{i}={\left(\sum_{j=1}^{M}{\left|{X}_{ij}-{X}_{j}\right|}^{2}\right)}^{1/2}.$$

Equation (1) computed the Euclidean distance of all samples from the test sample. Which M; is the number of features or inputs, Xij is the training sample, Xj is the test sample. Then, k minimum values of the obtained values for the vector D is selected for the next step. The output of the test point can be expressed by Eq. (2).

2 $${C}_{un}=\frac{1}{k}\sum_{t=1}^{k}{C}_{t}.$$

In Eq. (2), the value of C represents the label of the samples or the output value of the samples. This equation is used for KNN, but in WKNN, each coordinate axis is weighted according to its distance from the test data. The value of this weight is derived from Eq. (3):

3 $${w}_{i}=\frac{1/{D}_{i}}{{\sum }_{j=1}^{k}\left(1/{D}_{i}\right)} , i=\mathrm{1,2},\dots ,k.$$

In Eq. (3), the variable w is the weight for each of the k samples. In the following, the final value is calculated according to the weights using Eq. (4):

4 $${C}_{un}=\sum_{i=1}^{k}{w}_{i}{C}_{i}.$$

### Bee algorithm

The bee algorithm was developed in 2005. This algorithm simulates the feeding behavior in bee groups^17^. Bees can be divided into three categories: foraging bees and foraging bees. A bee that goes to a predetermined food source is called a worker bee, a bee that conducts a random search is called a foraging bee, and a bee that moves in the dance area to decide is called a foraging bee. Choosing a food source that is left over is called a fodder bee.

### Firefly algorithm

Fireflies are a kind of cockroaches that emit yellow and cold light in the process of bioluminescence. For various reasons (about which there is a difference of opinion, such as reproduction or creating a defense mechanism), night owls are more likely to move towards a night owl that is brighter than themselves. The distance of night lights from each other, the amount of ambient light absorption, the type of light source, and the amount of light emitted from the source are factors that affect the light received from a source.

The firefly algorithm is an optimization method which finds the optimal solution by simulating the behavior of the firefly^18–20^.

### Multilayer perception

Neural networks are intended to create patterns act as a human brain. The neural network works by creating an output pattern based on the input model delivered provided to the network^21,22^. Neural networks are composed of several processing elements or neurons that receive and process input data and ultimately provide an output from it. Input data may be raw data or the output of other neurons^23^. The output can be the final product or input for other neurons. An artificial neural network consists of artificial neurons, which are actually processing elements. Each neuron has several inputs and it assigned a weight to each input^24,25^. The average output of each neuron is obtained from the sum of all inputs multiplied by the weights. The final output is done by applying a transformation function.

Multilayer perception, or MLP, is an architecture of artificial neural networks in which it divided the neurons of the network into several layers^26^. In these networks, the first layer is the input and the last layer is the output, and the intermediate layers are called hidden layers^27^. This architecture can be called the most widely used architecture of neural networks.

### Hybrid methods

In this paper, it developed a hybrid method. This combined method results from the combination of several methods, such as FF, KNN, ABC, MLP and K-Means. In general, this combined method can be divided into three general parts and also has two phases of training and testing. To increase the prediction accuracy, we used the data of 8 wells and using the K-Means clustering method, we first put the wells that have similar behavior in one group, and then in the next block, using the data of the wells at time t and which group each well is in, the neural network estimates the output of the well for time t + 1.

**Figure 1:**
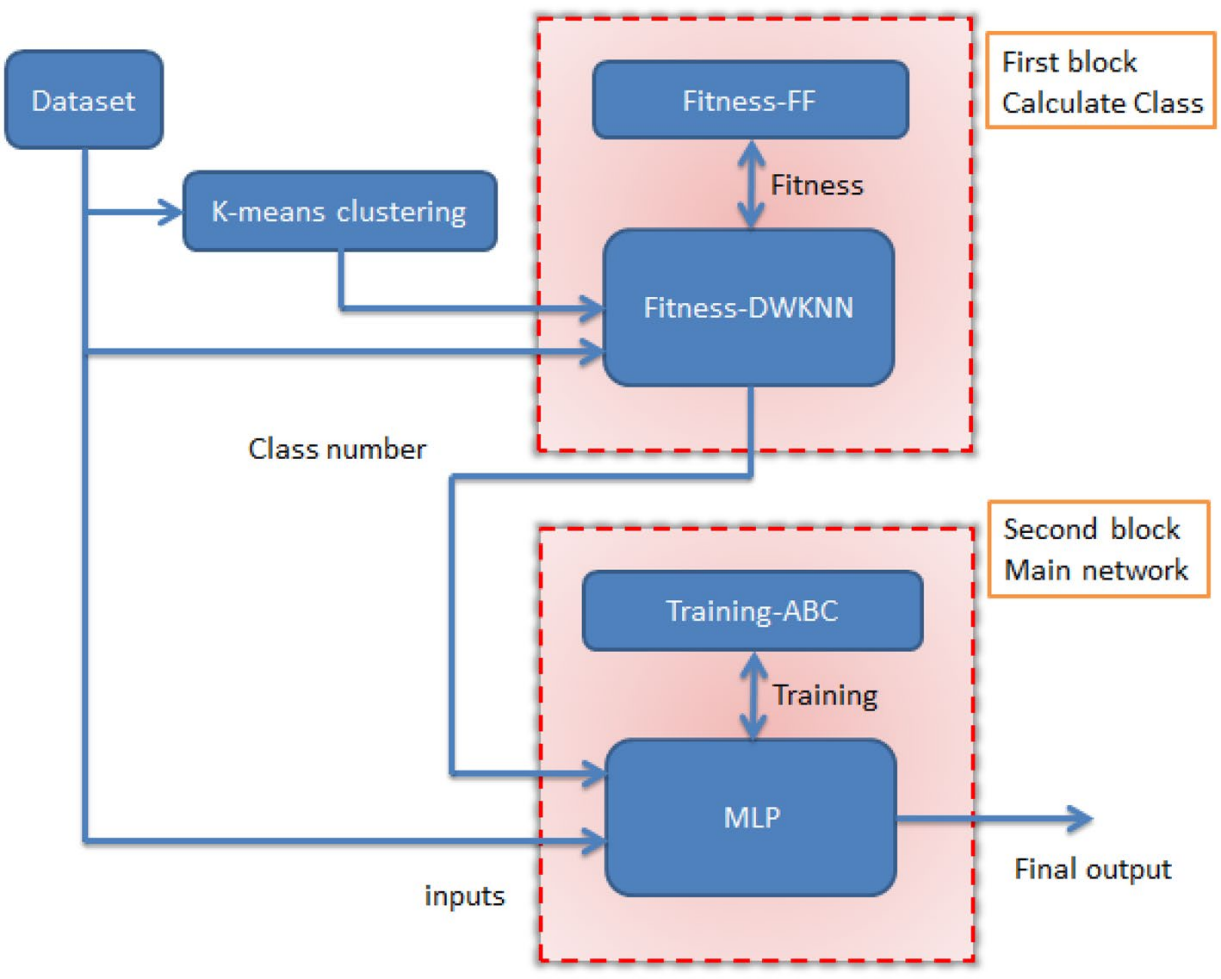
General flow chart diagram of the training stage of the new method.

In the new method, the KNN method is used as the basic method for classification and the FF optimization algorithm is used for find the optimal coefficients control’s parameter for the input data. In addition, MLP was used to estimate the output values, and we used the ABC algorithm for better training. To perform classification, we must use a new output value that we define ourselves. Therefore, we add a new output to the dataset and get its value using the K-Means algorithm. For the classification block, the input data is sent along with the new output. In the second step, when the classes are determined, the data is sent to the second block to estimate the value. In this block, the ABC-MLP combination is used to estimate the value. For this block, the input values are sent along with the new output. The control parameters for the approaches utilized in this article are listed in Table 1. Figure 1 shows the flow chart diagram of the training stage of the new method. The new method is made of two phases, training and testing, and we will first look at the training phase.

**Table 1:** Introduce control settings for new hybrid machine learning’s prediction of underground water level.

Parameter	Value	Parameter	Value
FF		ABC	
Max. iteration. no.	100	Max. iteration. no.	100
No. FF	50	No. bee	100
Coefficient-gamma	1	Source no.	50
Attraction coefficient	2	Onlooker no.	50
Mutation coefficient	0.2	Trial	60
Mutation coefficient damping ratio	0.98	Variables no.	3
Uniform mutation	0.05	MLP	
m	2	Input no.	5
Variables no.	3	Hidden layer no.	2
DWKNN		Input neurons no.	9
Mount of K	4	Hidden layer1 neurons no.	10
		Hidden layer2 neurons no.	5
		Output neurons no.	1
		Weights and biases no.	170

We have to use different data for each of the two phases. Therefore, we considered 70% of the data as training data for the training phase and the remaining 30% as test data for the test section. Based on the 70% of the data that we considered for the training part, we left 30% for validation.

### Training stage

First, the data should be normalized, which is done using Eq. (5).

5 $${x}_{i}^{l}=\frac{{x}_{i}^{l}-Min({x}^{l})}{Max\left({x}^{l}\right)-Min({x}^{l})}\times 2-1.$$

**Figure 2:**
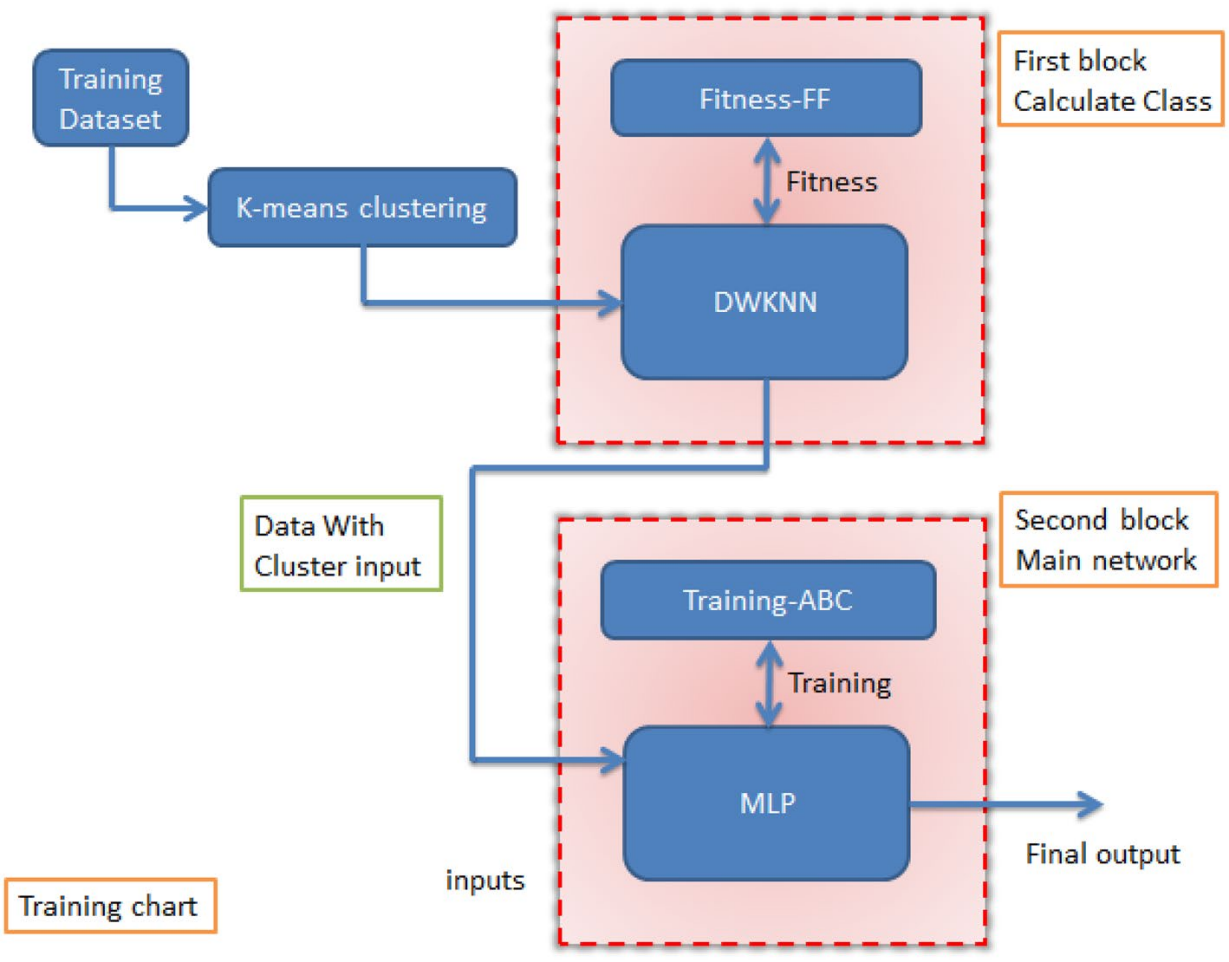
Flow chart diagram of the training stage of the new method.

In Eq. (5), variable M is the number of inputs, x_il_ is the lth input of the ith sample. The Max(xl) value is the lth largest input number and Min(xl) is the lth smallest input number. Figure 2 shows the block diagram related to the training stage.

After data normalizing, we have to add a new entry to the data, which specifies the number of the cluster or class to which each data belongs. We add that input to be used by the classification block and increase the accuracy of the estimate. Determining which class each data belongs it does to by the first block, but since the data is not labeled and not clustered, the number of classes and their data must be determined for this block first. Thus, using the K-Means block, we first determine the optimum number of classes and data for each class. The Davies Bouldin value was used to arrive at the optimum number of classes. The smaller this value is, the more optimal the number of classes is (Table 2).

**Table 2:** Determination of number of the cluster or class to which each data belongs.

Cluster number	2	3	4	5	6	7
Davies Bouldin value	6.4125	4.2135	5.2345	6.2456	7.2154	7.3654

**Figure 3:**
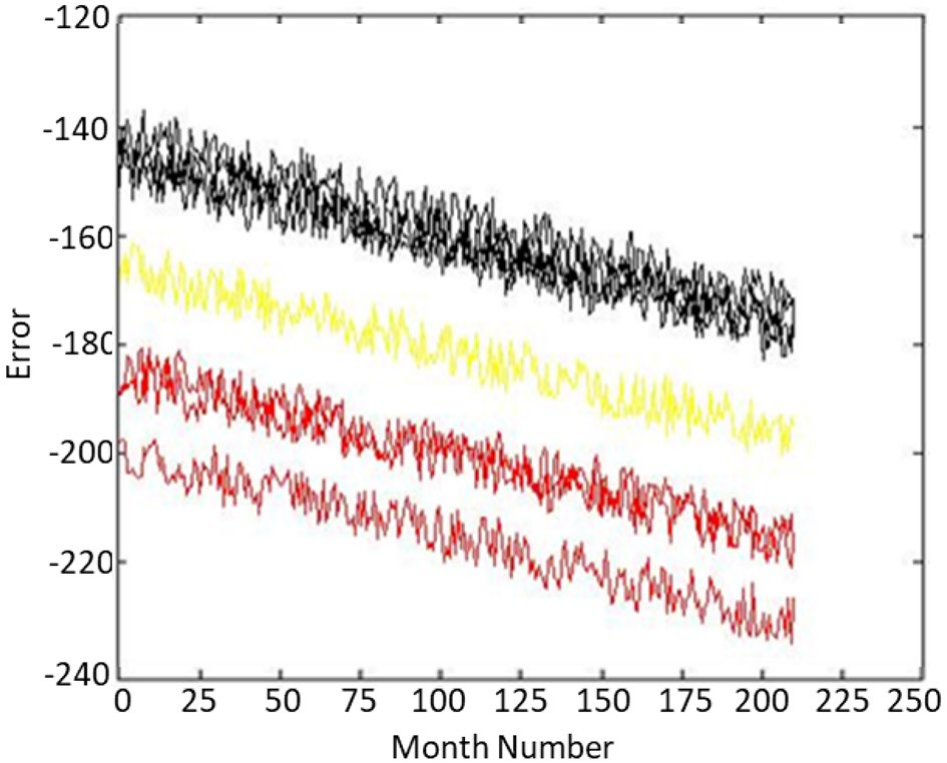
The data of 8 different wells which have been converted into three clusters and shown with three colours.

**Figure 4:**
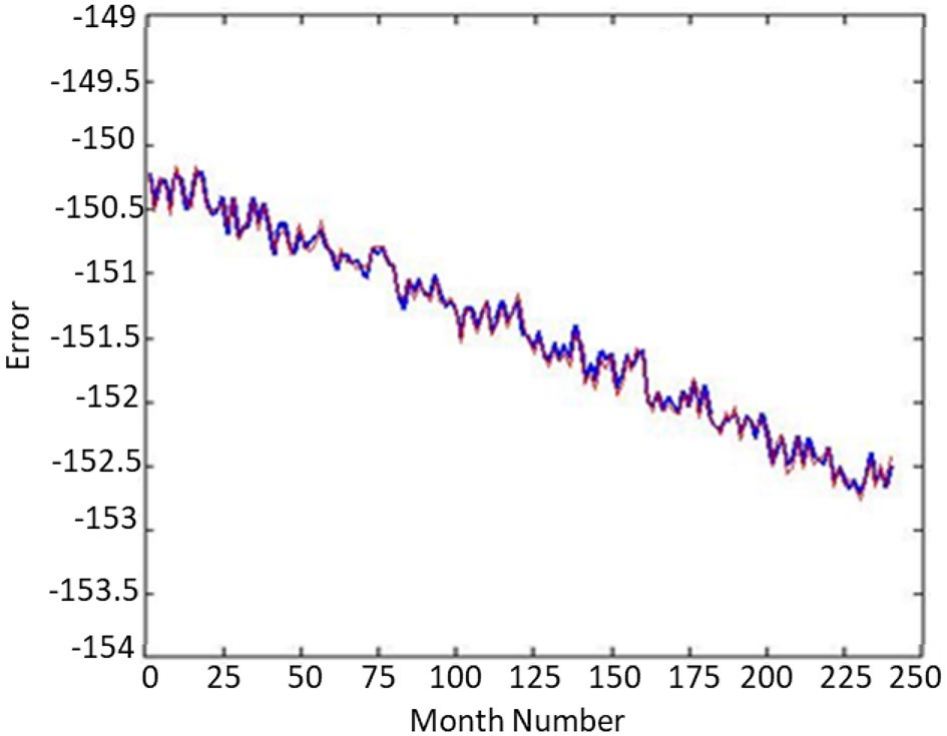
Validation results for well 1.

The smaller the Davies–Bouldin distance for a k, the more suitable the value of k is. Therefore, for the data of these 8 wells, the value is three clusters. Now, we divide all the data into three clusters using the K-Means algorithm and add a new output to each of the data, which stores the data class number and has values of one. It is up to three. In the Fig. 3, you can see the blocking of the wells. Figure 4 shows the well 1 validation. This graph displays the great accuracy of the algorithm’s results. Additionally, demonstrate how this technique may reduce noise in enormous data sets.

To create three clusters from the data of eight wells, we added a new column as the class number and assigned values between 1 and 3 based on the K-Means algorithm’s output. This operation only occurred during training, not during testing. The first block used data from outputs 1, 2, and 3 for categorization. To improve accuracy, we considered coefficients for each input using the firefly method to determine their best value. As optimization methods like firefly generate different solutions each time, they run due to a large number of optimal solutions, we obtained four weights with values of 0.1542987, 0.9254255, 0.4256712, and 0.6732144 from the algorithm's best answers for the four inputs. All inputs have an equal impact on output but may have different coefficients depending on their impact on output value.

### Error parameters

Equations (6) to (12) are given to determine the statistical comparing error of these algorithms. Based on the results presented in the results section and using these equations, we can compare the algorithms’ performance accuracy.

6 $${\mathrm{AE}}_{\mathrm{i}}=\frac{{\mathrm{S}}_{(\mathrm{Measured})}-{\mathrm{S}}_{(\mathrm{Predicted})}}{{\mathrm{S}}_{(\mathrm{Measured})}}\times 100,$$

7 $$\mathrm{MAE}=\frac{{{\sum }_{\mathrm{i}=1}^{\mathrm{n}}\mathrm{PD}}_{\mathrm{i}}}{\mathrm{n}},$$

8 $$\mathrm{MARE}=\frac{{\sum }_{\mathrm{i}=1}^{\mathrm{n}}\left|{\mathrm{PD}}_{\mathrm{i}}\right|}{\mathrm{n}},$$

9 $$\mathrm{STD}=\sqrt{\frac{{\sum }_{\mathrm{i}=1}^{\mathrm{n}}{({\mathrm{D}}_{\mathrm{i}}-\mathrm{Dimean})}^{2}}{\mathrm{n}-1}},$$

$$\mathrm{Dimean}=\frac{1}{\mathrm{n}}\sum_{\mathrm{i}=1}^{\mathrm{n}}\left({{\mathrm{S}}_{\mathrm{Measured}}}_{\mathrm{i}}-{{\mathrm{S}}_{\mathrm{Predicted}}}_{\mathrm{i}}\right),$$

10 $$\mathrm{MSE}=\frac{1}{\mathrm{n}}\sum_{\mathrm{i}=1}^{\mathrm{n}}{\left({{\mathrm{S}}_{\mathrm{Measured}}}_{\mathrm{i}}-{{\mathrm{S}}_{\mathrm{Predicted}}}_{\mathrm{i}}\right)}^{2},$$

11 $$\mathrm{RMSE}=\sqrt{\mathrm{MSE},}$$

12 $${\mathrm{R}}^{2}=1-\frac{\sum_{\mathrm{i}=1}^{\mathrm{N}}{({{\mathrm{S}}_{\mathrm{Predicted}}}_{\mathrm{i}}-{{\mathrm{S}}_{\mathrm{Measured}}}_{\mathrm{i}})}^{2}}{\sum_{\mathrm{i}=1}^{\mathrm{N}}{({{\mathrm{S}}_{\mathrm{Predicted}}}_{\mathrm{i}}-\frac{{\sum }_{\mathrm{I}=1}^{\mathrm{n}}{{\mathrm{S}}_{\mathrm{Measured}}}_{\mathrm{i}}}{\mathrm{n}})}^{2}}.$$

## Study area

The area under investigation is situated within the longitude range of 388,000 to 400,000 and the latitude range of 3,496,000 to 3,508,000. This region is located in the folded Zagros geological division of Iran and comprises anticlines and transects that vary in width, length, and height. The general orientation of this area is roughly northwest-southeast. The geological formations present in this region consist of rock units from the second and third ages as well as Quaternary sediments. The oldest rocks in this area are the thin limestones found in the Ilam-Soruk layer, followed by Pabdeh and Gurpi marl formations, Asmari limestone formations, chalk and marl layers from the Gachsaran formation, Bakhtiari conglomerate, and alluvial sediments arranged chronologically. The area being studied has two types of aquifers, alluvial and karst, from a hydrogeological perspective. The alluvial aquifer is located in the upper part of the Qalehtol plain and either reaches an impermeable bedrock or transforms into a karst aquifer at deeper levels. The karst aquifer is formed in the Asmari formation limestone and is limited by the impermeable Pabde formation below. There is no Gachsaran Formation outcrop on the northeastern side of the belt-long anticline, but on the southwestern side, it covers some areas of the Asmari Formation. Three limestone wells drilled by Khuzestan Water and Electricity Organization around the northwestern tip of Kamerdaraz anticline indicate a karst aquifer with high transfer and storage capabilities. The Asmari formation sinks under Barangerd plain from southeast to Qalehtol plain until it re-emerges in Haft Cheshme mountain north of Qalehtol. Two limestone wells with irrigation are also present in southeast Qalehtol plain. The northeastern edge of the anticline rises in Barangerd plain and finds a reversed state of syncline, while suspended sediment is enclosed on both sides by Pabdeh Formation, and in Tang Kurd, limestone outcrop represents termination of the aquifer.

## Data base

The underground water level, which determines the level of fresh underground water and is used for drinking water and other applications including agriculture, etc., depends on various parameters such as the underground water level (for the previous three consecutive years), rainfall It depends on rain, river discharge and harvest discharge.

In order to predict the underground water level, it collected 2112 data points using artificial intelligence hybrid algorithms from information related to 8 wells in an area of Khuzestan province (Qale-Tol area). This information includes the flow rate of the river entrance (feeding fresh water resources), underground water level, rainfall and underground water withdrawal by examining different time delays, as well as the level of underground water during the years 1992 to 2013. Is the important point in these data is that to determine the output (determining the underground water level for time t), the information of the input parameters related to time t, t-1 and t-2 has been used.

The statistical parameters related to 8 wells are reported in Table 3 for the groundwater level (m), rainfall (mm), river rate (m^3^/s) and discharge rate (m^3^/s), respectively, for the information related to 8 wells from 1992 to 2013. Based on this model, it is possible to determine the parameters of the underground water level as a function of the parameters of the underground water level (for the previous three consecutive years), rainfall, river discharge and harvest discharge (Eq. 13);

**Table 3:** Determining statistical parameters for underground water level, rainfall, river discharge and harvest discharge for the information related to 8 wells related to the years 1992–2013.

Statistical parameters	Well-1	well-2	Well-3	Well-4	Well-5	Well-6	Well-7	Well-8
Groundwater level (m)								
Mean	152.45	137.45	137.45	141.46	170.45	176.45	144.45	142.45
Std. deviation	1.28	1.28	1.28	1.29	1.27	1.27	1.27	1.27
Variance	1.63	1.63	1.63	1.65	1.61	1.60	1.62	1.61
Minimum	150.21	135.23	135.17	139.23	168.21	174.21	142.22	140.23
Maximum	154.69	139.70	139.72	143.69	172.69	178.69	146.69	144.69
Rainfall (mm)								
Mean	9.99	9.99	10.00	10.02	10.01	10.01	10.01	9.99
Std. deviation	11.26	11.26	11.26	11.29	11.28	11.27	11.28	11.25
Variance	126.22	126.29	126.34	126.90	126.70	126.64	126.70	126.15
Minimum	0.00	0.00	0.00	0.00	0.00	0.00	0.00	0.00
Maximum	34.31	34.32	34.32	34.42	34.34	34.34	34.35	34.32
River rate (m^3^/s)								
Mean	2860.17	2860.12	2860.18	2859.15	2860.35	2859.85	2860.36	2871.15
Std. deviation	223.13	223.20	223.12	222.46	222.93	222.82	223.57	224.92
Variance	49,600.29	49,630.06	49,595.63	49,301.99	49,508.33	49,459.95	49,796.33	50,396.98
Minimum	2344.52	2344.89	2344.61	2340.57	2353.68	2340.36	2344.73	2344.89
Maximum	3351.84	3352.13	3353.71	3359.47	3356.40	3355.70	3363.61	3359.47
Discharge rate (m^3^/s)								
Mean	5460.17	5460.20	5460.10	5460.69	5459.29	5459.83	5461.11	5436.49
Std. deviation	254.29	254.37	254.24	255.31	255.12	254.45	253.80	236.72
Variance	64,420.23	64,457.93	64,394.83	64,936.20	64,840.30	64,498.05	64,170.35	55,822.83
Minimum	5031.11	5028.83	5032.21	5014.59	5020.65	5025.23	5020.63	5028.83
Maximum	5888.82	5889.46	5893.33	5907.05	5904.51	5901.38	5904.27	5888.22

13 $${Q}_{t}=f\left({R}_{t-2},{R}_{t-1}, {R}_{t},{P}_{t-2},{P}_{t-1},{P}_{t},{O}_{t-2},{O}_{t-1}{O}_{t}\right).$$

In this equation, Q = groundwater level, R = rainfall, P = river rate and O = discharge rate.

**Figure 5:**
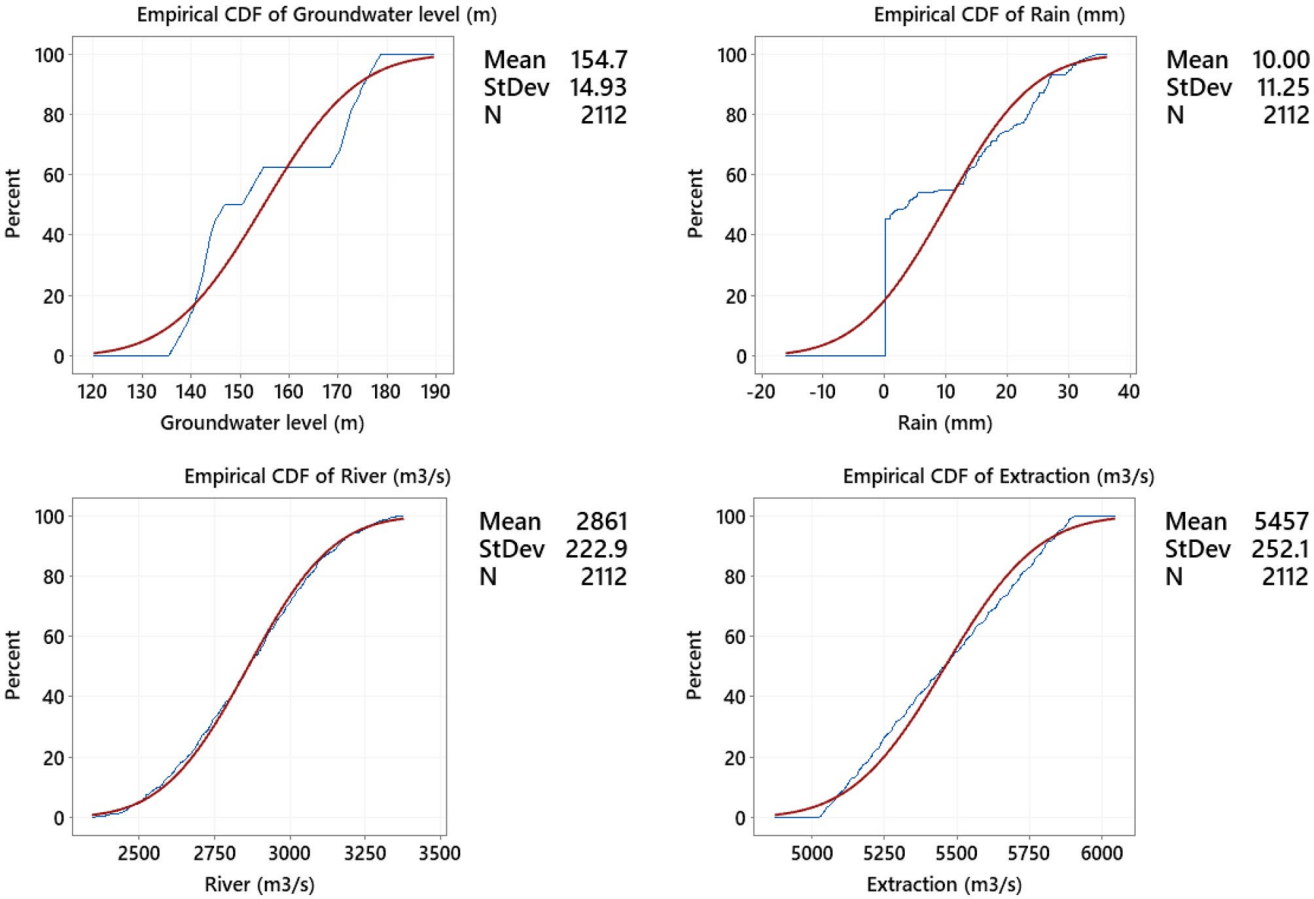
Cumulative distribution function diagram for the variables of groundwater level, rainfall, river rate and discharge rate.

The method used to describe input and output data in scientific articles is the use of cumulative distribution functions. It also used this method in this article to describe the data. It described information about the distribution of 2112 data points:

Figure 5 shows information about normal distribution functions for predicting the groundwater level. The value of cumulative distribution function for groundwater level (Q) as 140 is approximately 16% and for 16 < Q < 154 it is approximately 45% and for the rest of the data this value is approximately 39% for Q > 154. For rainfall (R) as 12, it is approximately 56%, and for 12 < R < 14, it is approximately 6%, and for the rest of the data, this value of R > 14 is approximately 38%. For river rate (P) as 2852, it is approximately 48%, and for 2852 < P < 3207, it is approximately 46%, and for the rest of the data, this value of P > 3207 is approximately 6%. For discharge rate (O) as 5115, it is approximately 10%, and for 5115 < O < 5476, it is approximately 41%, and for the rest of the data, this value of O > 5476 is approximately 49%. As it is clear in Fig. 5, the data related to river discharge and harvest discharge have a normal distribution, and the data related to rainfall and underground water level are non-normally distributed.

## Result and discussion

As mentioned before, the aim is to predict the underground water level from 2112 data points and using artificial intelligence hybrid algorithms from the information related to 8 wells in one region of Khuzestan province. The recombinant hybrid artificial intelligence algorithms used are FF-KNN, ABC-KNN and DL-FF-KNN-ABC-MLP algorithms. To develop algorithms and test them, we used well information from wells 1–5, and for their development, we used well information from wells 6–8. In order to developed these algorithms, 70% of the data related to 5 wells (wells 1–5) was used as training and 30% of this data was used as testing (in order to make a proper comparison between the algorithms, a similar train and test sub set have been used).

And also, in order to check information related to artificial intelligence algorithms, error statistical parameters have been used, and with this statistical metric, it can make a correct comparison between the algorithms used in this thesis in order to predict the underground water level. The results related to training, testing and the total data used (well data 1–5) to determine this valuable index are reported in Tables 4, 5 and 6, respectively (based on Eqs. (6) to (12)).

**Table 4:** Determination of statistical errors for the training data related to the information related to wells 1–5 (70% of this data set).

Models	MAE	MARE	STD	MSE	RMSE	R^2^
DL-FF-KNN-ABC-MLP	− 0.002	0.033	0.060	3.56E−03	0.0597	0.9999
FF-KNN	0.004	0.116	0.236	5.54E−02	0.2354	0.9989
ABC-KNN	0.000	0.247	0.405	1.64E−01	0.4051	0.9968

**Table 5:** Determination of statistical errors for test data related to information related to wells 1–5 (30% of this data set).

Models	MAE	MARE	STD	MSE	RMSE	R^2^
DL-FF-KNN-ABC-MLP	0.001	0.025	0.045	2.03E−03	0.0451	0.9999
FF-KNN	0.000	0.051	0.092	8.44E−03	0.0919	0.9990
ABC-KNN	0.012	0.243	0.445	1.98E−01	0.4448	0.9970

**Table 6:** Determination of statistical errors for the entire data set related to the information related to wells 1–5 (100% of this data set).

Models	MAE	MARE	STD	MSE	RMSE	R^2^
DL-FF-KNN-ABC-MLP	− 0.001	0.030	0.054	4.92E−03	0.0701	0.9999
FF-KNN	0.003	0.090	0.191	6.10E−02	0.2471	0.9990
ABC-KNN	0.005	0.245	0.421	2.96E−01	0.5441	0.9969

The results presented for different algorithms for test data, training and the whole data set are given in Tables 4, 5 and 6. One aim of this treatise is to compare and present new algorithms of FF-KNN, ABC-KNN and DL-FF-KNN-ABC-MLP algorithms for predicting the underground water level. According to the results presented in the table reports, the performance accuracy of DL-FF-KNN-ABC-MLP for predicting this key parameter is very high. According to the reports shown in Tables 4, 5 and 6, it is RMSE_Train_ = 0.0451, RMSE_Test_ = 0.0597 and RMSE_Total_ = 0.0701. Moreover, on the basis of the results presented and the comparison between STD, a good comparison of the performance accuracy of the algorithms can be made. In other words, the comparison for this term shows that the accuracy of the algorithms for predicting underground water level is ABC-KNN < FF-KNN < DL-FF-KNN-ABC-MLP.

**Figure 6:**
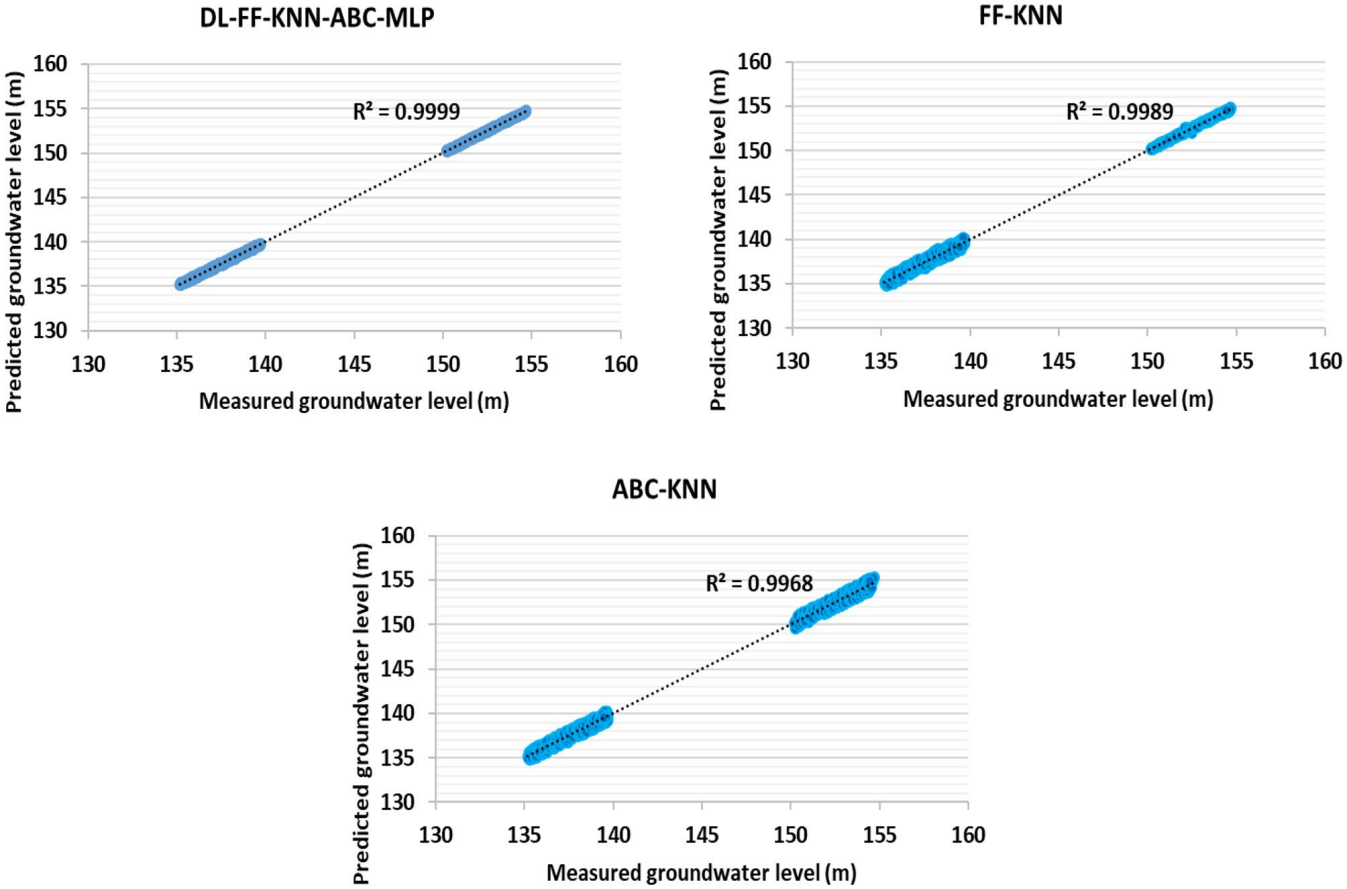
Cross-plot for groundwater level prediction for training data related to information related to wells 1–5 (70% of this data set).

**Figure 7:**
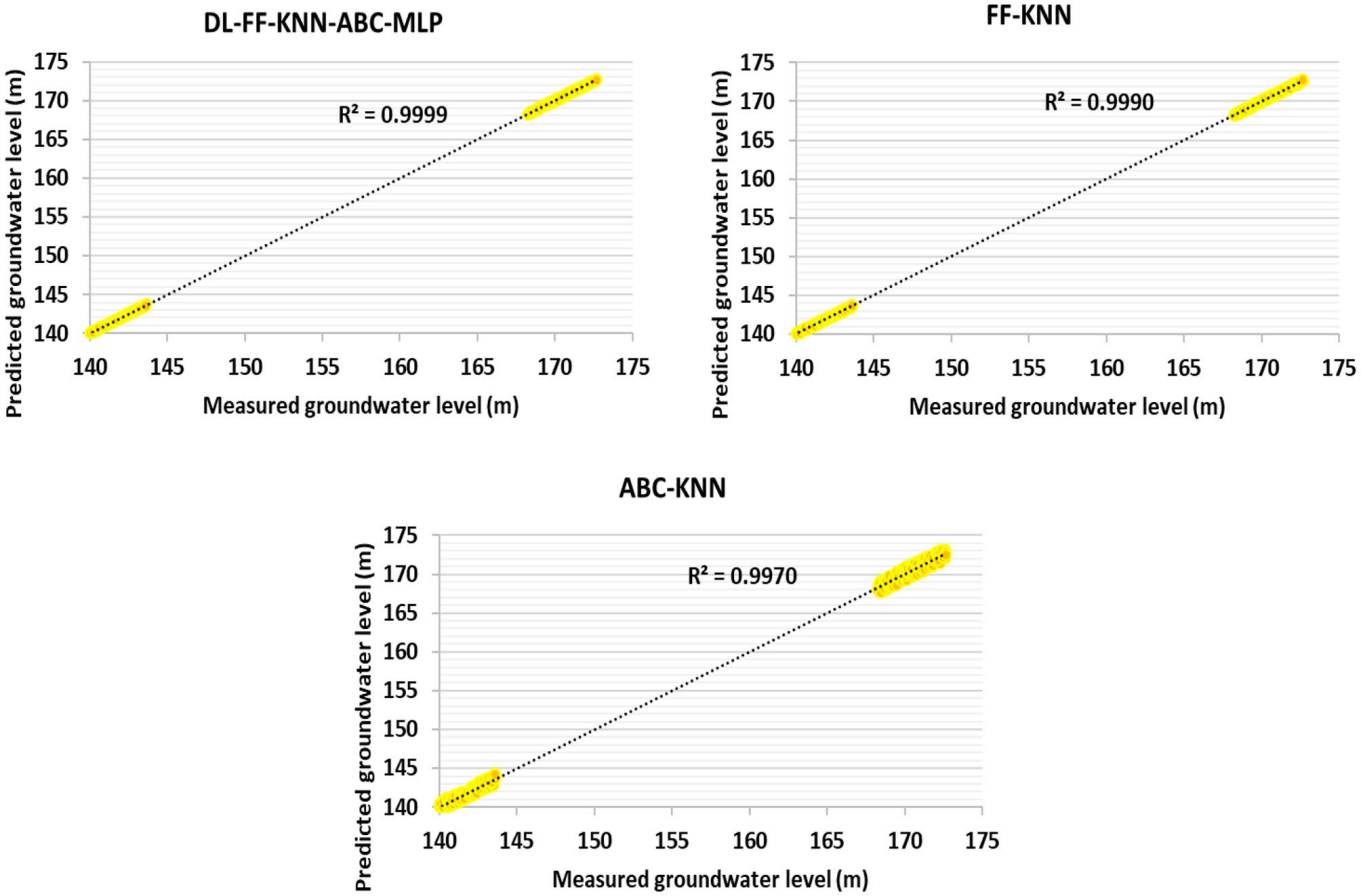
Cross diagram for groundwater level prediction for test data related to information related to wells 1–5 (30% of this data set).

**Figure 8:**
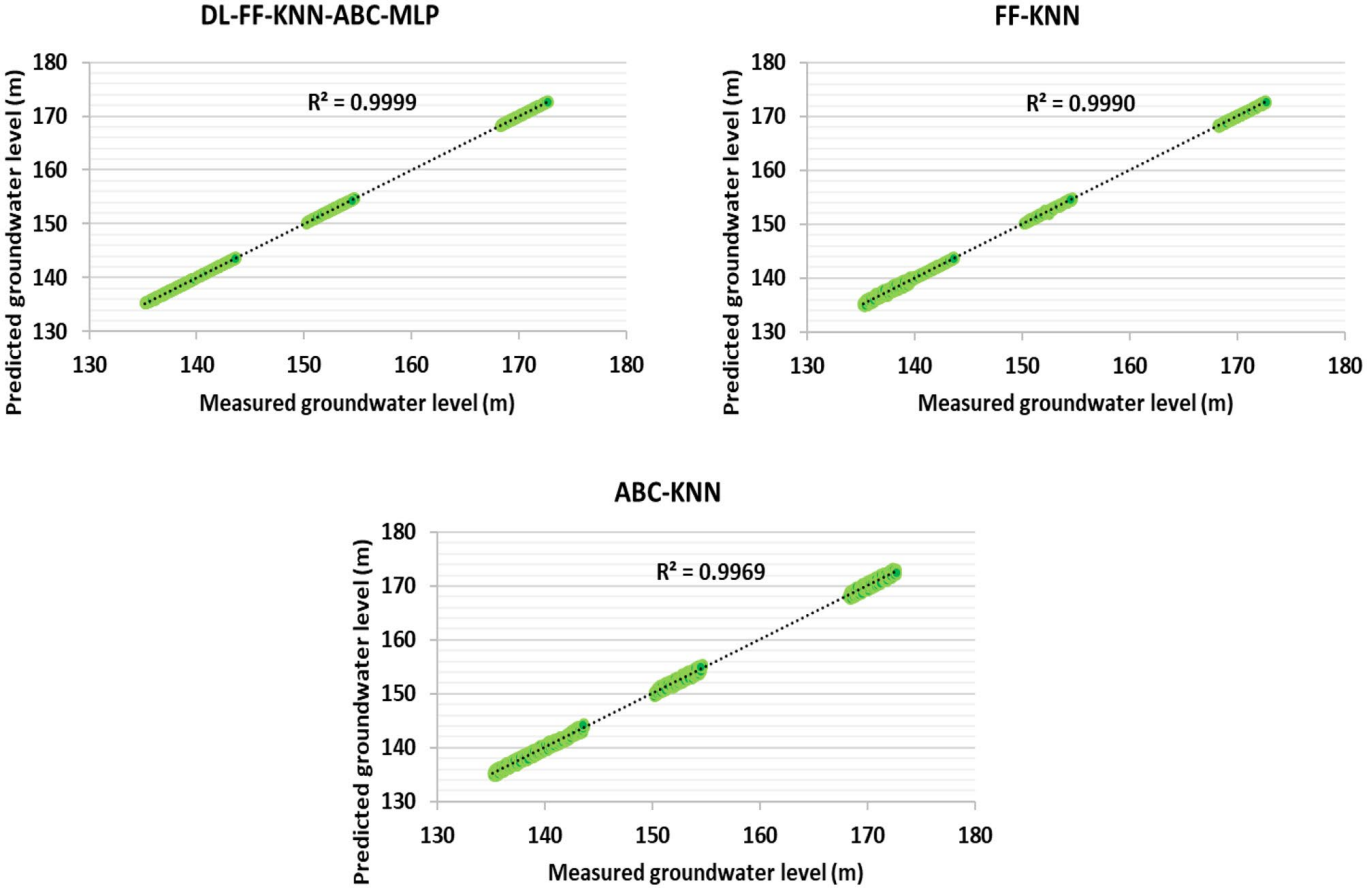
Cross-plot for predicting the groundwater level for the entire data set related to the information related to wells 1–5 (100% of this data set).

Figures 6, 7 and 8 show the cross-plots for the groundwater level to check the predicted data against the measured data, respectively, for the data related to training, testing and the entire data set. One of the important and practical statistical errors which can determine the algorithm performance is the use of the R-square statistical error. With these data, you can understand the accuracy of the functions and also check the data using graphical diagram. As shown in this figure, the R-square value for the DL-FF-KNN-ABC-MLP algorithm has the highest performance accuracy. Based on Figs. 6, 7 and 8, using the cross line, the performance accuracy of the predicted points against the measured points can be measured by using the distance of these points with the cross line. Based on Figs. 6, 7 and 8 which show training, testing, and total, it is clear that the distance of points with the cross line for hybrid models is ABC-KNN > FF-KNN > DL-FF-KNN-ABC-MLP.

## Development of the new model

**Figure 9:**
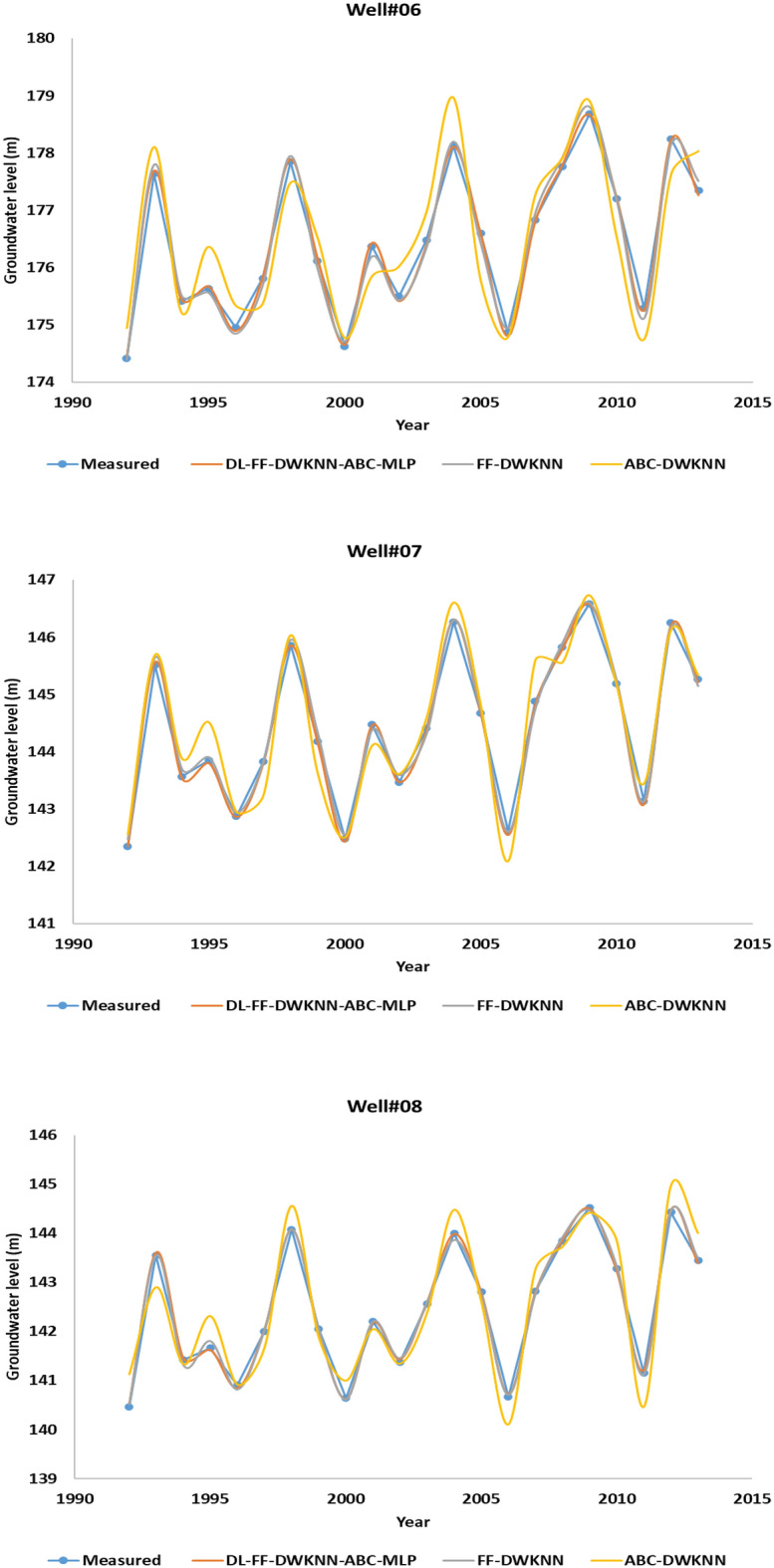
Comparison for groundwater level prediction for the entire data set for wells 6, 7 and 8 based on ABC-KNN, FF-KNN and DL-FF-KNN-ABC-MLP.

This section of the article discusses the development and comparison of various models, including ABC-KNN, FF-KNN, and DL-FF-KNN-ABC-MLP (shown in supplementary file), for predicting groundwater levels in different wells in the same field. The study used information related to wells 6, 7, and 8 and first tested the models on wells 1–5, followed by checking the models on the remaining wells. Figure 9 provides a comparison of groundwater level predictions by year for wells 5 to 8 for the algorithms. The results show that the DL-FF-KNN-ABC-MLP algorithm outperformed the other algorithms in terms of performance accuracy for predicting groundwater levels in new wells in the same field. The study suggests that this algorithm could also be used in other fields and for predicting other key factors. The use of new information highlights the potential for this algorithm to be applied in various scenarios, and future researchers are encouraged to explore its application in other fields.

## Conclusion

In this study, 2112 data sets collected from 8 wells were used to predict the underground water level of Khuzestan region in Iran (Qale-Tol area). In order of prediction this parameter, three new artificial intelligence hybrid algorithms FF-KNN, ABC-KNN and new developed hybrid DL-FF-KNN-ABC-MLP algorithm have been used. Variable data which used as input data for hybrid machine learning, includes the flow rate of the river (which feeds fresh water sources), the level of underground water, precipitation and withdrawal of underground water by examining the delay of different times and also the level of underground water level during the years are 1992 to 2013. In order to developed these algorithms, 70% of the data related to 5 wells (wells 1–5) was used as training and 30% of this data was used as testing. The results show that the performance accuracy of the DL-FF-KNN-ABC-MLP algorithm is better than the other two algorithms used in this article. The novelty of this technique is that it first does classification by presenting the first block (combination of FF-DWKNN algorithm) and predicts with the second block (combination of ABC-MLP algorithm). The algorithm's ability to decrease data noise will be enabled by this feature. The results shown for this algorithm for the data related to testing, training and the entire data set are RMSE_Train_ = 0.0451, RMSE_Test_ = 0.0597 and RMSE_Total_ = 0.0701. It is suggested that other scientists use this modified algorithm to determine important parameters in the prediction of other hydrological parameters. In addition, it is suggested that scientists use the term reservoir temperature and soil moisture effect to predict groundwater levels. Also, researchers can use this algorithm for big data with high noise.

## Supplementary Information

Supplementary file.

## Data Availability

Based on the correct academic requirement, corresponding author will let to available to dataset.
